# Nematocidal activity of zinc oxide nanoparticles synthesized using chicken egg albumin in lung and spleen of *Parascaris equorum* experimentally infected rats

**DOI:** 10.1038/s41598-025-26546-4

**Published:** 2025-11-26

**Authors:** Sara Bayoumi Ali, Ayman Saber Mohamed, Sohair R. Fahmy, Manal El–Garhy, Mohamed R. Mousa, Fathy Abdel-Ghaffar

**Affiliations:** 1 Zoology Department, Faculty of Science, Cairo University, Giza, Egypt; 2 Pathology Department, Faculty of Veterinary Medicine, Cairo University, Giza, Egypt

**Keywords:** Parascaris equorum, Nanoparticles, Zinc, Nematodes, Embryonation, Horses, Diseases, Drug discovery, Medical research, Microbiology

## Abstract

Weight loss, weakness, coughing, nasal discharge, and neurological problems result from *Parascaris equorum* infection. However, severe infestations can cause intestinal impaction, colic, unthriftiness, peritonitis, and small intestine rupture, resulting in death. Parasite resistance to conventional drugs is mainly caused by decreased drug absorption and increased metabolism. Nanoparticle-based drugs can be viable alternatives. The study studied the pharmacological effects of zinc oxide nanoparticles( ZnO NPs) on *P. equorum*, infected rats’ lungs and spleens. Thirty-six male rats were separated into two groups of 18 rats each: infected and uninfected. The two groups were divided into three subgroups that received orally distilled water, 30 mg/kg ZnO NPs, or 60 mg/kg for 10 days. Depending on dosage, ZnO NPs (30, 60 mg/kg) drastically reduced larvae in the lung and spleen. ZnO nanoparticles reduced malondialdehyde and nitric oxide and improved the antioxidant system in sick rats’ spleens and lungs. The infected lung and spleen showed fewer histological changes after ZnO NPs (30, 60 mg/kg) therapy. Compared to rats infected but untreated, ZnO NPs at 30 and 60 mg/kg reduced nuclear factor-kappa B levels in immunohistochemistry. ZnO NPs do not harm animal cells and are harmless to non-infected rats. They can treat *P. equorum* infection due to their anthelmintic, antioxidant, healing, and anti-inflammatory properties.

## Introduction

A common parasitic worm that infects young horses and other equids like zebras, donkeys, and mules is called *Parascaris equorum*. For young horses, especially those under two years old, the nematode is extremely dangerous^1^. Symptoms of a *P. equorum* infection include neurological problems, weight loss, coughing, nasal discharge, and weakness. On the other hand, severe infestations cause peritonitis, colic, intestinal impaction, unthriftiness, and finally mortality from small intestine rupture^2^. The adult worm of *P. equorum* spends its three-month direct life cycle in the small intestine, where it generates a massive number of eggs.

Transmission of *P. equorum* occurs when an infected equid sheds eggs in feces. Once an equid ingests infective eggs, larvae hatch and migrate from the small intestine to the liver, then to the lungs, and finally back to the small intestine via the trachea esophageal route^3^. The worms mature and reproduce in the small intestine, then begin the life cycle again. Larval migration can be highly harmful, causing lesions in the liver, lungs, and bronchial and hepatic lymph nodes^4^. In the absence of an effective vaccine, chemotherapy becomes the primary effective tool to control helminth parasites. There are three classes of anthelmintic drugs: Benzimidazoles (fenbendazole and febantel), tetrahydropyrimidines (pyrantel), and macrocyclic lactones (ivermectin and moxidectin)^5^. Generally, the development of parasite resistance to conventional drugs results from decreased drug uptake and increased metabolism of the drug.

Therefore, the development of effective alternatives is essential, which can be achieved by nanoparticle-based drug formulations. In order to reduce the incidence of the parasite infection in locations where it is widespread, new, economical, and effective treatment options are needed. According to^1^, zinc oxide nanoparticles, or ZnO NPs, have shown great potential as dependable and efficient treatments for parasitic illnesses. Zinc oxide nanoparticles (ZnO NPs) have been demonstrated by^6^ to possess antibacterial, antifungal, antimicrobial, antioxidant, anthelmintic, and antiprotozoal properties. Recently, researchers have shown that the biosynthesis method of NPs has more advantages over physical and chemical synthesis^7^. The main drawbacks of physical and chemical processes for synthesizing nanomaterials are using toxic compounds, consuming a high amount of energy, and generating hazardous wastes. However, the green synthesis of nanoparticles is considered an eco-friendly technology because it does not involve poisonous compounds and is safe in handling^8^. In the current study, green-synthesized zinc oxide nanoparticles were synthesized from egg white. According to^9^, chicken egg albumin (CEA) exhibits remarkable physicochemical and biological properties, including excellent foaming, gelling, emulsifying, and nutritional capabilities, as well as high water solubility and the ability to form complexes with metal ions. Acting as a strong and biocompatible capping and reducing agent, egg albumin contributes to nanoparticle stability through its diverse functional groups—carboxyl, hydroxyl, and amino—which facilitate effective coordination with metal ions, guiding nucleation and growth while preventing aggregation. This work aimed to assess the anthelmintic and therapeutic effects of environmentally friendly synthesized zinc oxide nanoparticles (ZnO NPs) on experimentally infected rats’ *P. equorum* in the lungs and spleen.

## Materials and methods

### Synthesis of ZnO NPs using egg albumin

After adding 30 ml of chicken egg albumin (CEA) to 70 ml of Zn (CH_3_CO_2_)_2_ (0.25 M), the solution was agitated for 20 min. In order to induce precipitate, a pH 7.0 ammonia solution was introduced, then the sample was centrifuged at 1957 x g for a duration of 10 min. The particle underwent a three-hour process of washing, drying, and sintering at 400 °C in a vacuum oven^10^.

### Purification of ZnO NPs synthesized using egg albumin

Purification of the prepared nanomaterials was conducted using a volumetric mixture of methanol, hexane, and isopropanol in a ratio of 1:5:1. ZnO NPs were added to methanol to produce ZnO methanol colloids. When hexane and isopropanol were added to ZnO methanol colloids, the precipitation of white ZnO nanoparticles occurred. The solution was kept at 0 °C overnight until the ZnO NPs precipitated in their entirety. Following centrifugation and supernatant removal, the precipitated ZnO was agitated by hand to recombine it with methanol^11^. reported that the procedures above were iterated multiple times until all impurities were eliminated from ZnO NPs and a clear supernatant was obtained.

### Characterization of ZnO NPs synthesized using egg albumin

Different methods were applied for the characterization of the prepared nanoparticles.

#### X-ray diffraction (XRD) analysis

Using XRD with Cu–Kα radiation, the dimensions and crystallographic characteristics of ZnO nanoparticles were analyzed (XPERT–PRO, PANAnalytical). Using Scherrer’s formula, the average crystallite diameter (d) of the particles was calculated as follows: d equals K/β cosδ. In the given system, K represents the crystallite shape factor (0.89), λ denotes the X-ray wavelength of Cu Kα radiation (0.154 nm), θ signifies the Bragg diffraction angle, and β signifies the corresponding diffraction peak’s full width at half maximum^7^.

#### Transmission electron microscope (TEM)

The dimensions and morphology of ZnO nanoparticles were ascertained by employing electron microscopy at an accelerated voltage of 120 kV (JEM-JEM 2100 F; JEOL Ltd, Tokyo, Japan) at the Electron Microscopy Unit, Faculty of Agriculture, Cairo University^12^.

#### Ultra-violet–visible (UV-Vis) spectroscopy

The characterization of ZnO NPs was confirmed by measuring the absorbance (A) in UV–Vis spectrophotometer (Shimadzu UV-1601) at a wavelength range between 200 and 700 nm at room temperature and operated at an interval of 10 nm^12^.

#### Fourier transform infrared (FT‑IR) analysis

The functional groups of ZnO NPs were analyzed using Fourier transform infrared spectroscopy (FT-IR) to evaluate any chemical interactions that may have occurred between ZnO NPs and other components. The ZnO NPs were freeze-dried, combined with a potassium bromide (KBr) pellet, and then pressed into a disk shape. After that, an FT-IR spectrophotometer (JASCO FTIR-6200, JASCO International Co., Ltd.-Japan) was used to record IR spectra (range 4000–400 cmˉ1)^12^.

### Collection of *P. equorum* adults

An examination for parasite infestation was conducted on the gastrointestinal tracts of recently slaughtered donkeys (Equus ferus caballus Linnaeus 1758, Family Equidae) at the Faculty of Veterinary Medicine, Cairo University. Isolated from an infected donkey, adult female worms were preserved in Goodwin’s solution at 37 °C until required.

### Egg embryonation

**Fig. 1:**
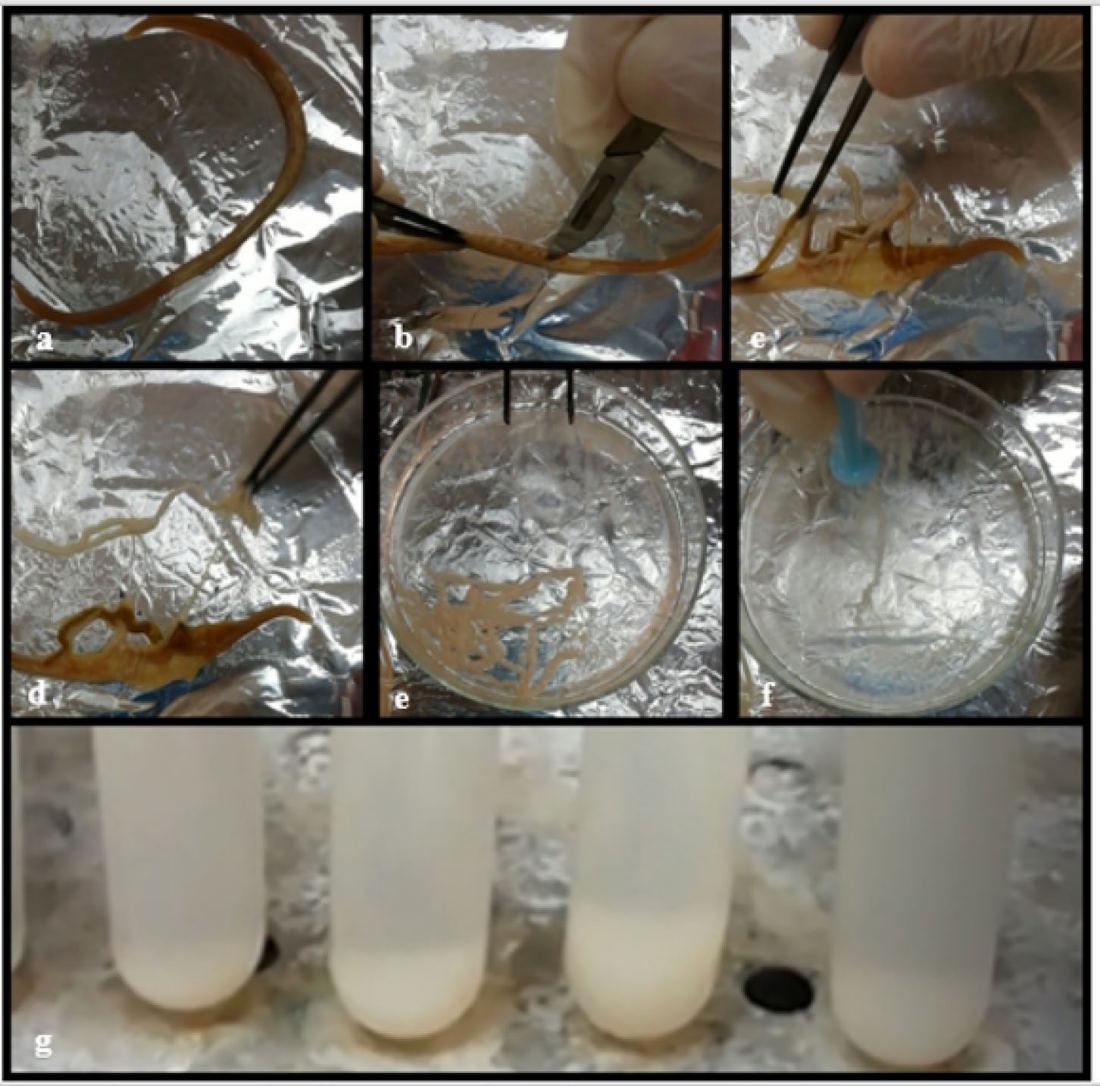
General steps for egg preparation.

Eggs were obtained after dissection of female worms in acidified water (pH = 3) and concentrated by centrifugation at 176 x g for 5 min. Twenty days of incubation were spent in 0.5% formalin for embryonic development. Twice daily, this egg solution was manually agitated to promote the development of third-stage larvae. The embryonated eggs were subsequently rinsed three times with saline to eliminate the formalin solution^4,13^ (Fig. 1).

### Animals in experimentation

Male Wistar rats, ranging in weight from 150 to 160 g, were procured from the Dokki branch of the National Research Center (NRC) in Giza. The rats were confined in polypropylene enclosures within an animal house with adequate ventilation. The rodents were maintained in a controlled environment between 22 and 25 degrees Celsius with a 12-hour light-dark cycle. Seven days were allotted for acclimation to the milieu before the experiment. At all times, water and standard chow pellets were made readily accessible to the rodents.

### Acute toxicity study

A total of eight male rats were used in the LD50 determination. Four categories of animals were designated as follows: A-D. ZnO NPs were administered orally at doses of 10, 100, 300, and 600 mg/kg to each group. Following administration, the rats were monitored for one hour, with subsequent assessments conducted every two hours for twenty-four hours, in search of indications of toxicity such as fatigue, loss of appetite, and paw licking, as well as mortality. The LD50 was calculated utilizing the following equation:

where M_0_ is the highest dose of biocompatible ZnO NPs that caused no mortality; M_1_ is the lowest dose of ZnO NPs that caused mortality^14^.

### Experimental design

**Table:** 

Groups	36 male rats					
Duration	Group A (18 non-infected rats)			Group B (18 infected rats)		
	Subgroup I (Control)	Subgroups II	Subgroups III	Subgroup I (Vehicle)	Subgroups II	Subgroups III
1 st day	Saline			1000 *P. equorum* eggs [15]		
For 10 days	Distilled water	ZnO NPs (1/10 LD50)	ZnO NPs (1/20 LD50)	Distilled water	ZnO NPs (1/10 LD50)	ZnO NPs (1/20 LD50)

### Care of animals and the collecting of specimens

Euthanasia is part of our study. It was done by using a single intraperitoneal dose of sodium pentobarbital (50 mg/kg) as an anesthetic agent immediately after the animals were exsanguinated (cutting a major blood vessel). The lung and spleen samples were extracted, rinsed with physiological saline, and divided into three equal portions. The first sample was utilized for histopathology and immunohistochemical evaluations, the second sample was used for *P. equorum* larval retrieval, and the third sample was employed for the oxidative stress experiment.

### Larval recovery from lung and spleen tissues

The acid-isolation approach was used to extract *P. equorum* larvae from lung and spleen tissues. More precisely, a fixed amount of infected organs (1 g) was mixed with 2 ml of a hydrochloric acid solution with a concentration of 0.5%. The specimens were subjected to incubation at a temperature of 37 °C for a period of 24 h in order to promote the sedimentation of larvae. Following the incubation period, the tissues that were infected were extracted, and the larvae present in the HCL solution were separated by centrifugation at a low speed for 5 min. The liquid portion was extracted, and the solid portion containing the immature form of an organism was observed using a microscope that uses light to magnify the image^15^.

### Preparing tissue homogenates

Tissues from the lung and spleen were weighed and homogenized (10% w/v) in 0.1 M Tris-HCl buffer, which was chilled to a pH of 7.4. The homogenate was centrifuged at 704 x g for 10 min at 4 °C, and oxidative stress indicators were measured in the supernatant that was left behind.

### Oxidative stress markers

Biodiagnostic kits (Giza, Egypt) were used to evaluate the levels of malondialdehyde (MDA), catalase (CAT), nitric oxide (NO), reduced glutathione (GSH), and glutathione-S-transferase (GST) in the lung and spleen homogenate supernatant in accordance with the manufacturer’s instructions.

### Histopathology analysis

Hematoxylin and eosin (H&E) staining was performed on lung and spleen samples after they were embedded in paraffin, fixed in 10% neutral-buffered formalin, and sectioned.

### Immunohistochemistry analysis

For immune staining, lung tissue sections were sectioned and placed on adhesive glass slides. In brief, tissue transparencies were subjected to peroxidase blocking after epitope retrieval. Following a two-hour incubation at room temperature with primary anti-NF-κβ at a dilution of 1:100, the slides were rinsed with phosphate buffer saline. The reaction was generated and observed in accordance with the manufacturer’s guidelines using a Mouse/Rabbit Immuno-Detector DAB HRP reagent (Bio SB, CA, USA). Negative control samples were generated through the omission of the primary antibody procedure. The area percent of positive expression was determined utilizing the Cell Sens Dimensions Olympus software, developed by Olympus in Tokyo, Japan^16^.

### Statistical analysis

Statistical analysis utilized one-way analysis of variance (ANOVA) and the least significant difference (Duncan) post hoc test to compare group means. Data are presented as mean ± SE, with *p* < 0.05 considered significant.

## Results

### Characterization of ZnO NPs synthesized using egg albumin

#### X‑ray diffraction analysis

**Fig. 2:**
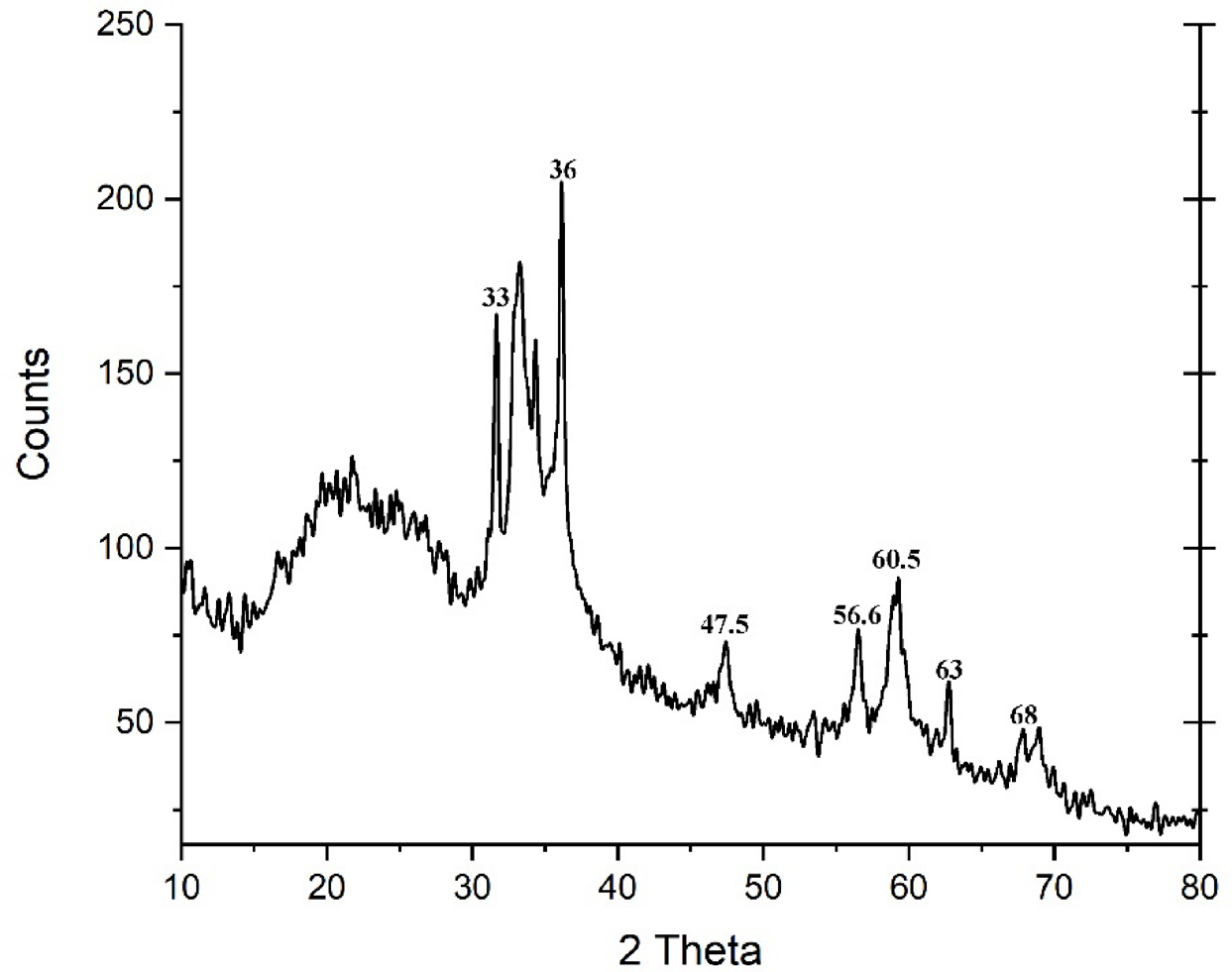
XRD Pattern of ZnO NPs synthesized using egg albumin.

XRD determined the crystalline structure of ZnO NPs. The peaks at 2 θ = 33°, 36°, 47.5°, 56.6°, 60.5°, 63°, and 68° correspond to the crystal plans (0 0 2), (1 0 1), (1 0 2), (1 1 0), (1 10), (1 0 3), and (2 0 2) of ZnO NPs. According to the diffraction peaks, the crystal structure of ZnO NPs resembled hexagonal zincite. It was determined that the mean size of ZnO NPs was approximately 20 ± 2 nm (Fig. 2).

#### Transmission electron microscope (TEM)

**Fig. 3:**
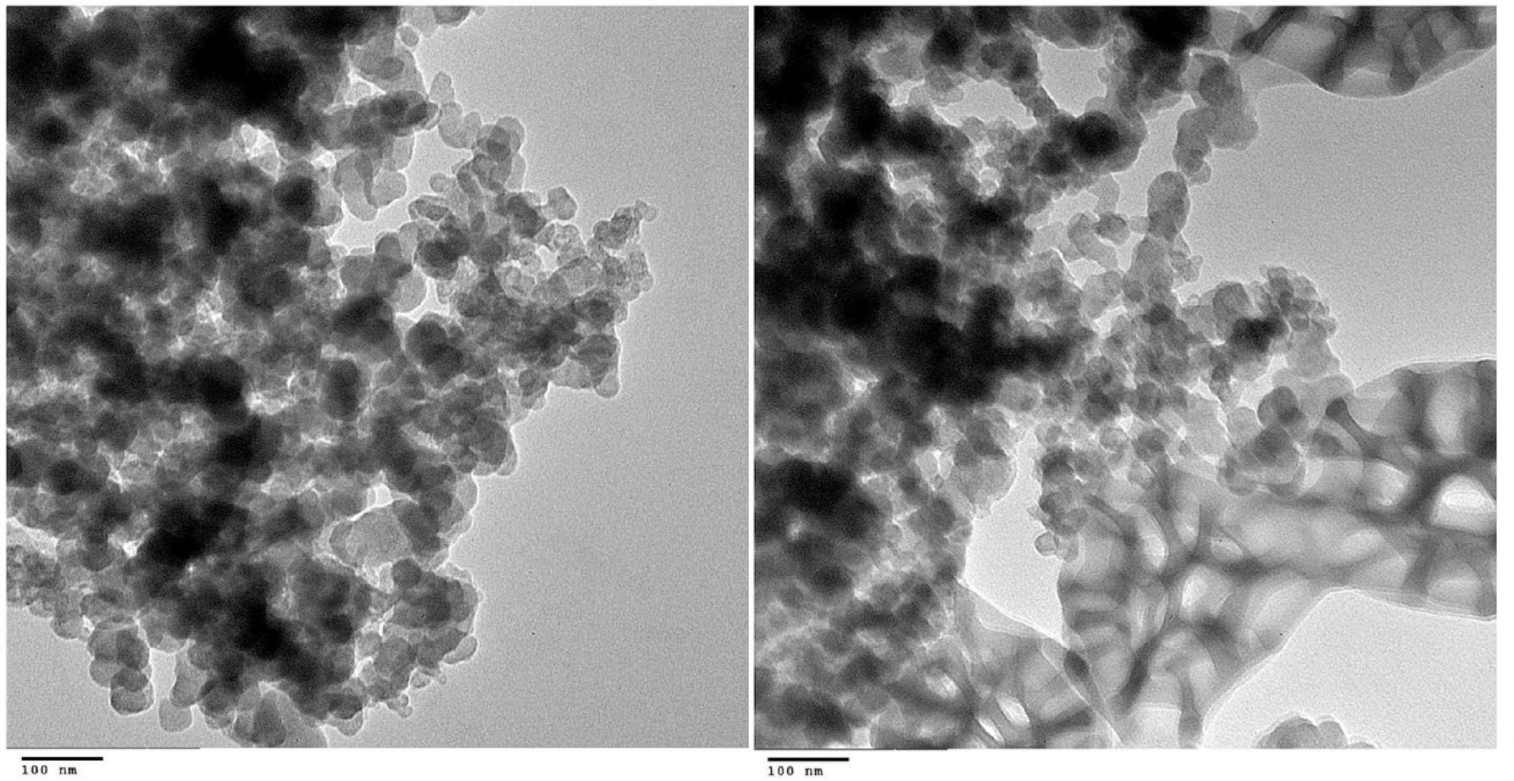
TEM image of ZnO nanoparticles synthesized using egg albumin.

A confirmation of the XRD result was the polygonal morphology, smooth surface areas, and average diameter of 21 nm of the preponderance of the ZnO nanocrystals (Fig. 3).

#### UV–Vis spectroscopy

**Fig. 4:**
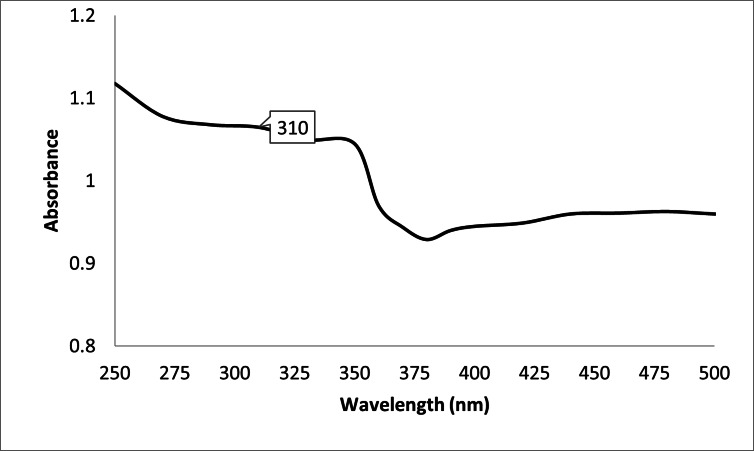
UV-Vis spectroscopy of ZnO nanoparticles synthesized using egg albumin.

Characteristic peaks of green-synthesised ZnO NPs were seen in their UV spectra between 250 and 350 nm. At 310 nm is the sample characteristic peak (Fig. 4).

#### Structural characterization of ZnO NPs FT‑IR spectra

**Fig. 5:**
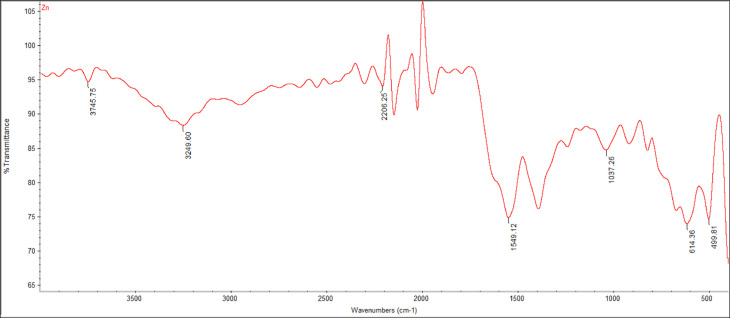
FTIR spectra absorption peaks of ZnO NPs synthesized using egg albumin.

Figure 5 showed FTIR spectra absorption peaks in the range of 500–4000 cm − 1 centered at 3745.75 cm − 1 that matched with the stretching modes and vibration of intermolecular hydrogen bonds (O–H), revealing the hydroxyl groups. The N–H stretch was found at 3249.6 cm − 1, and close to aliphatic primary amine, Alkyne (CΞC stretch) at 2206.25 cm − 1. The nitro compound (N-O stretch) was at 1549.12 cm − 1, anhydride CO-O-CO stretch band was found at 1037.26 cm − 1, halo compound C-Cl stretch band was found at 614.3 cm − 1, and the characteristic peak at 499.61 cm − 1 indicated the formation of the stretching mode of ZnO NPs.

### ZnO NPs acute toxicity

ZnO NPs were found to be lethal up to a dosage of 600 mg/kg. The suggested LD50 from the acute toxicity research served as the basis for selecting the median effective dose (ED50). Selected doses are 1/10 and 1/20 of the 600 mg/kg body weight suggested LD50.

### Morphological study of non-embryonated and embryonated *P. equorum* eggs and larvae

#### Light microscopic examination of eggs

**Fig. 6:**
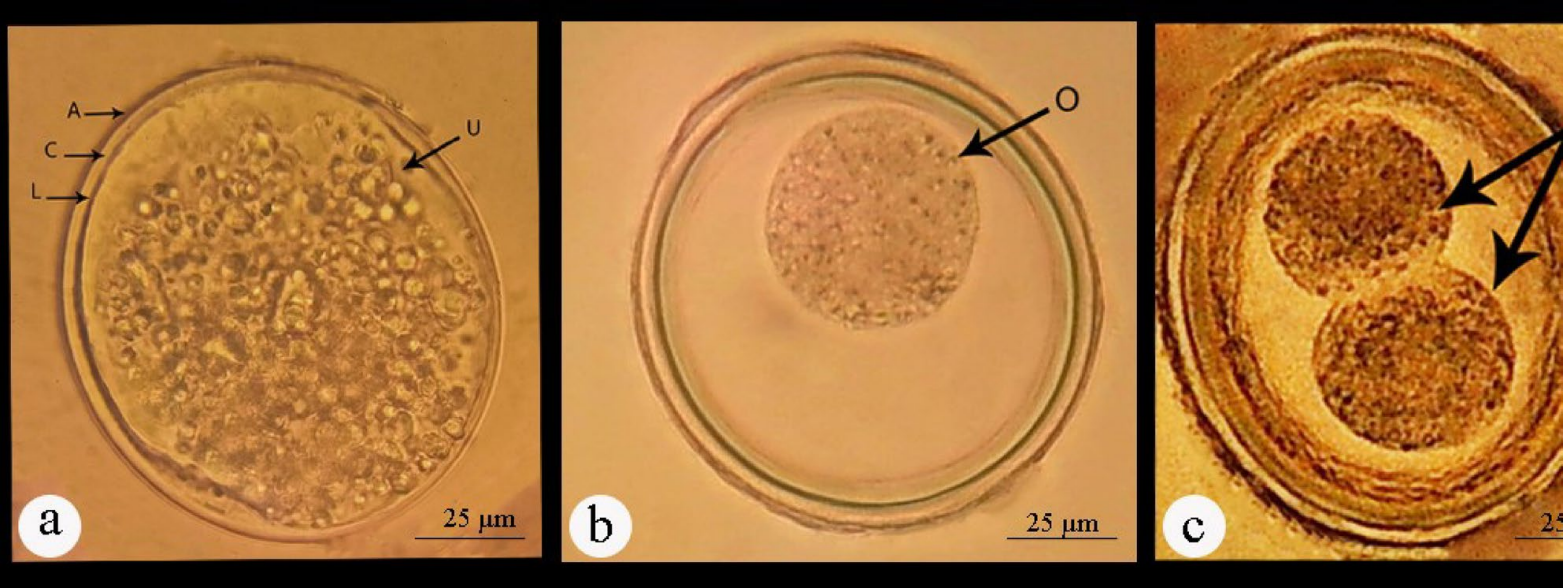
Photomicrographs of non-embryonated *P. equorum* eggs. **(a**). Freshly isolated egg from the uterus of an adult female surrounded by the albuminous layer (A), chitinous layer (C), and lipid layer (L), and containing undeveloped unicellular ovum (U). (**b**). Developed egg showed one-cell-stage (O), (**c**). Developed egg showed two-cell-stage (T).

**Fig. 7:**
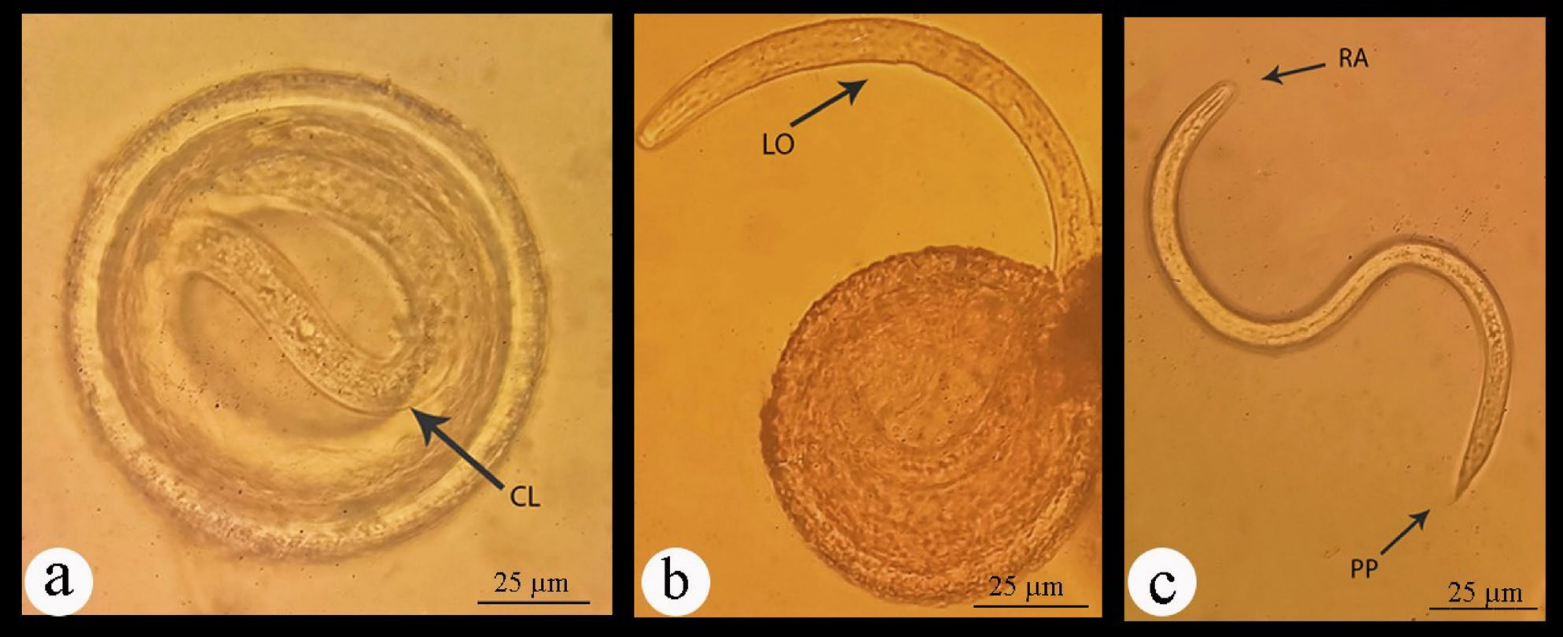
Photomicrographs of embryonated *P. equorum* eggs. **(a**). Embryonated egg possessing a coiled larva (CL). (**b**). Hatching egg showing a larva coming out (LO). c. Free larva with rounded anterior (RA) and pointed posterior (PP) ends.

**Fig. 8:**
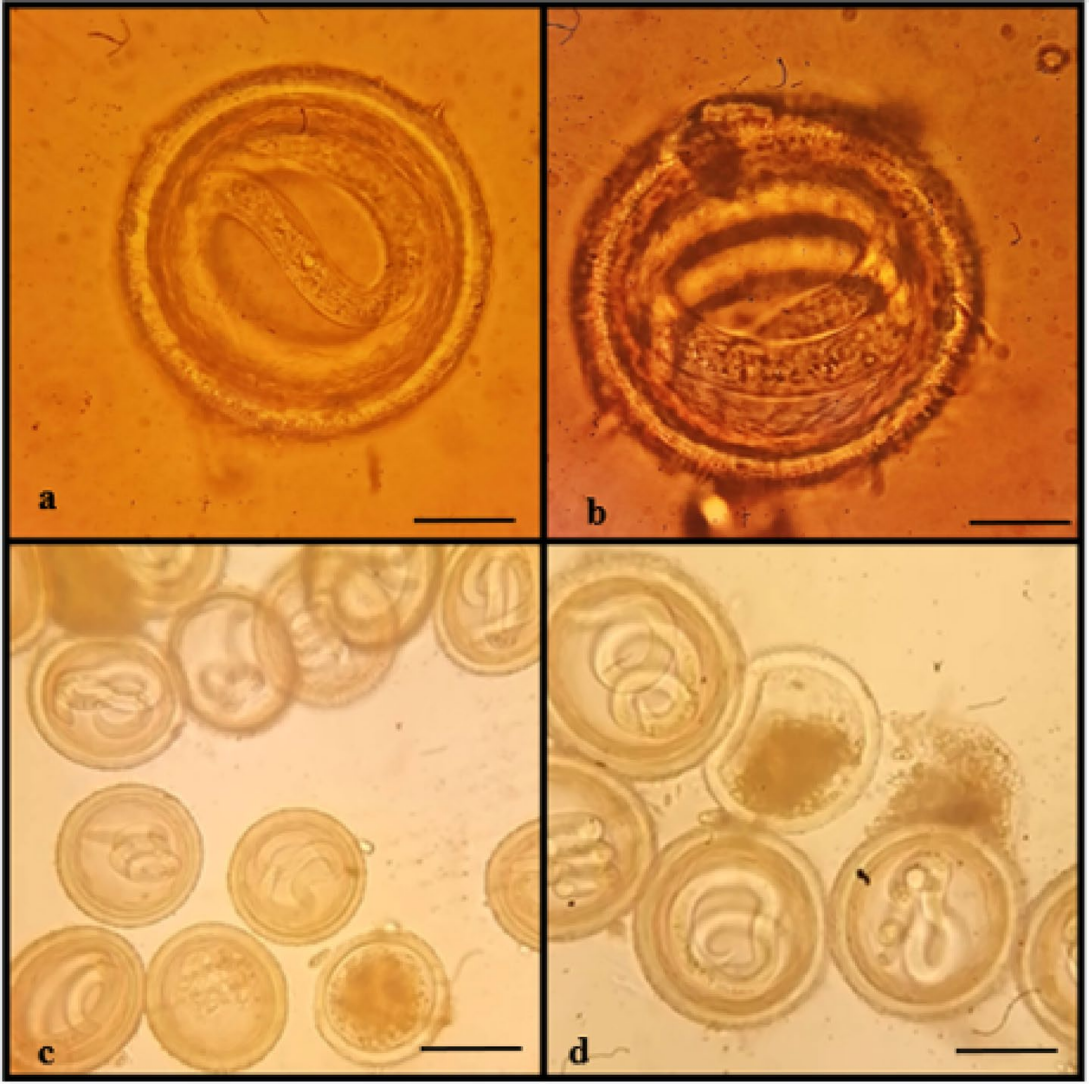
Photomicrographs of embryonated *P. equorum* eggs.

The nematode eggs are oval in shape and covered by three layers. The external protein (albuminous) layer is exogenous in origin and deposited on the outer surface of the egg by the uterus wall after fertilization. The middle or the true shell layer is the thickest layer secreted by the egg itself and is composed of chitin fibers tightly associated with proteins. The chitinous layer is semipermeable but usually rigid and serves as an enclosure to protect the developing larva from physical trauma. The innermost layer contains lipids in all nematodes and protects the larva from osmotic and desiccation threats, toxins, and anthelmintics. The chitin content in the eggshell varies in shape and thickness among different species of nematodes. The external margin of the protein layer may be mammillated or smooth. The mammillations vary greatly, appearing obtuse, undulate, or irregular in shape. The present study described the different developmental stages of *P. equorum* eggs. They were more spherical in shape when compared to other nematode eggs. Non-embryonated eggs had an undeveloped (unsegmented) unicellular ovum. While embryonated eggs contained one to two individual cells (1 and 2 cell stages) (Fig. 6). Well-developed eggs had a fully grown and distinguishable larva that was very active at a warm temperature. In a mature egg, the larva was actively mobile and coiled once or twice within the shell. The emerged larva was slender, cylindrical in shape, with a rounded anterior and pointed posterior ends (Figs. 7 and 8).

#### Scanning electron microscopy (SEM) examination of eggs

**Fig. 9:**
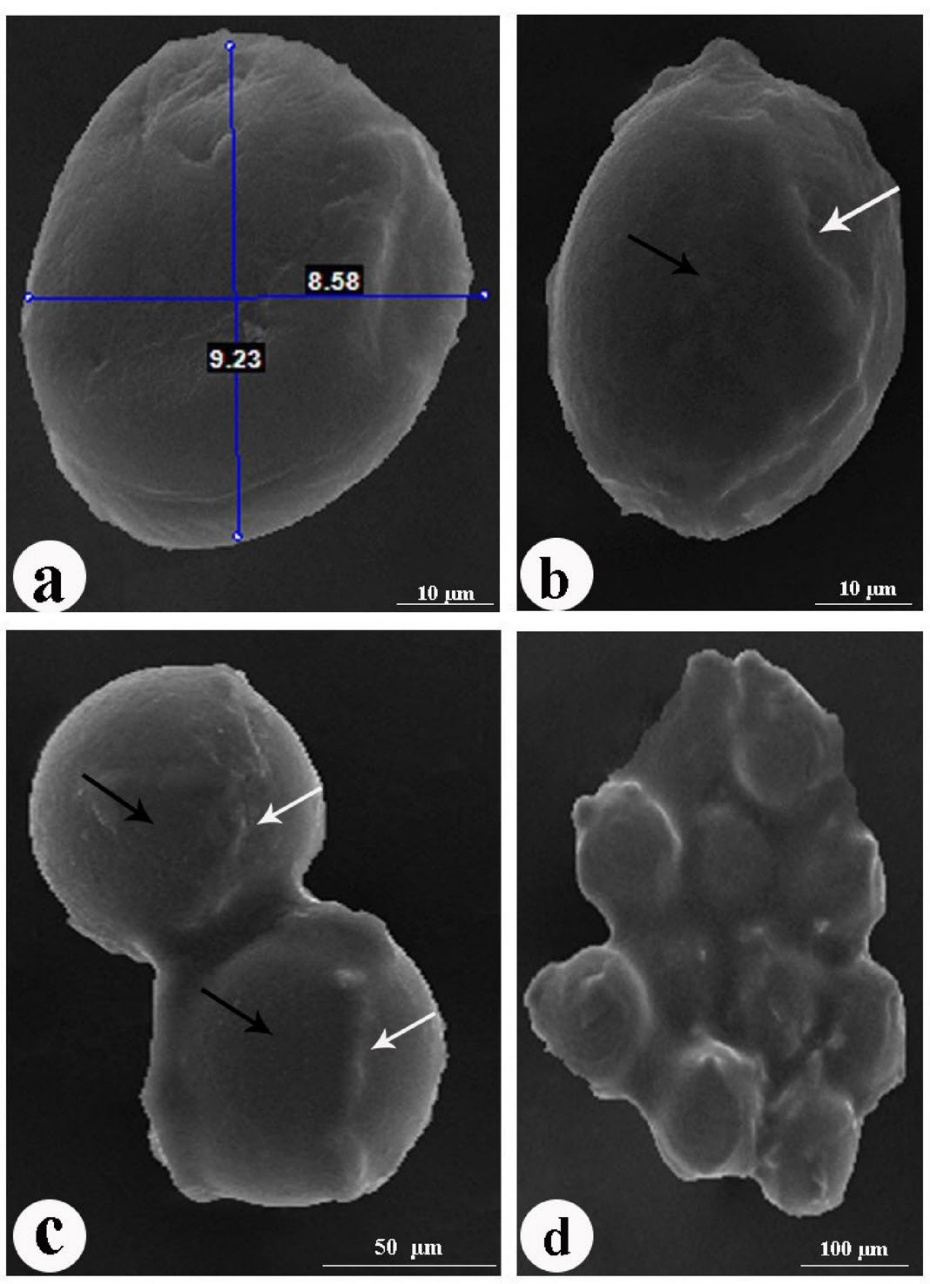
Scanning electron micrographs of non-embryonated *P. equorum* eggs. **(a**,** b**). Non-embryonated eggs had a smooth covering (black arrow) with a plateau (white arrow). c, d. Clusters of non-embryonated eggs with a plateau (white arrow).

Figure 9 showed scanning electron micrographs of non-embryonated eggshell ultrastructure. The non-embryonated eggshell appeared to be slightly oval in shape, measuring 60–65 μm in length. The non-embryonated eggshell had an appropriately smooth surface with ridges and plateaux.

**Fig. 10:**
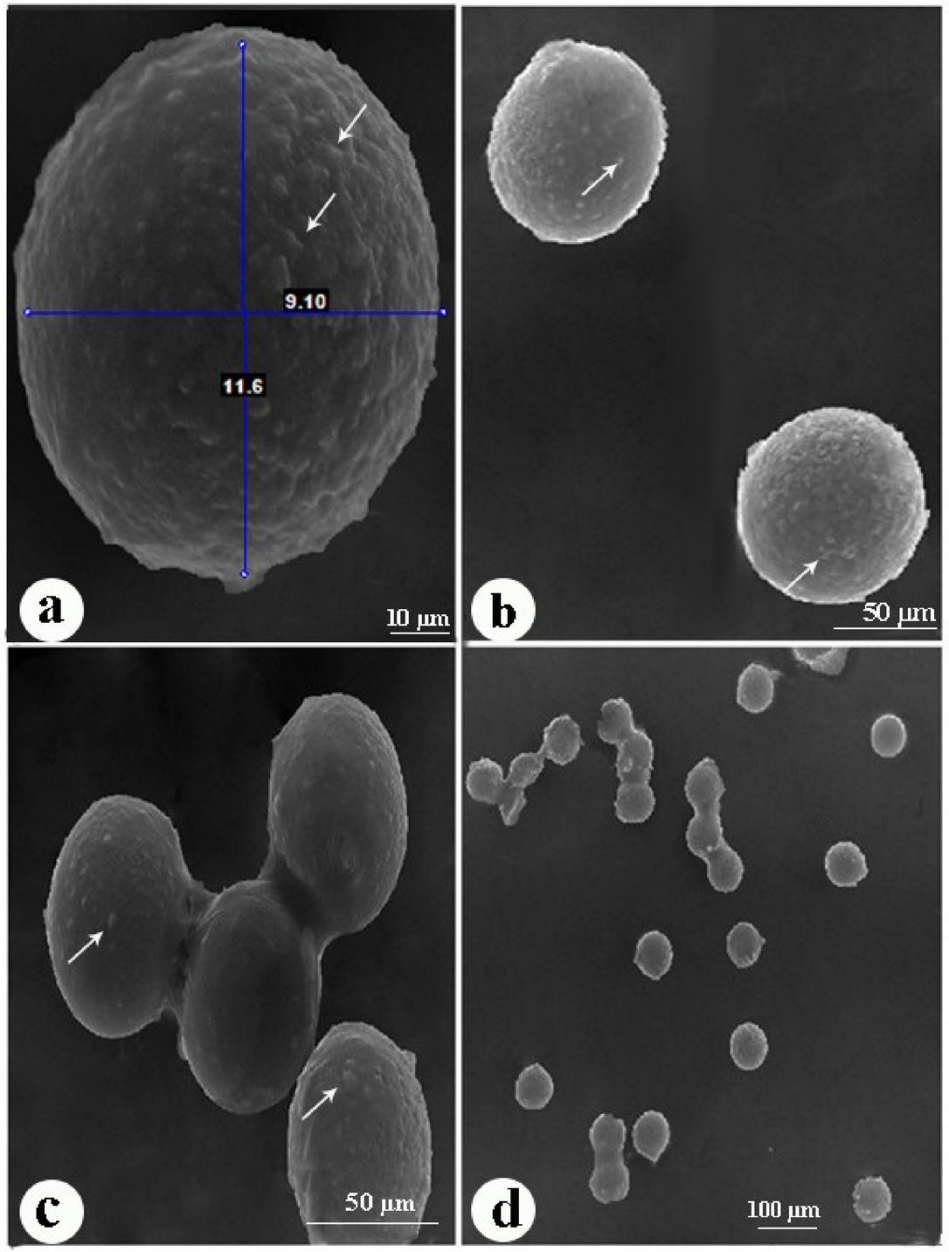
Scanning electron micrographs of embryonated *P. equorum* eggs. (**a**,** b**). Embryonated eggs had a granular covering (arrow). c, d. Clusters of embryonated eggs.

Figure 10 showed scanning electron micrographs of embryonated eggshell ultrastructure. The embryonated eggshells were larger in size and rounder in shape than the non-embryonated eggshell, measuring 80–85 μm in length. The surface of the embryonated eggshell appeared granular and devoid of plateaux and ridges.

The non-embryonated eggs had a strong tendency to cluster with one another, which led to the formation of egg clumps, especially those isolated directly from the uteri of female worms (Fig. 9c, d). The embryonated eggs had a lower tendency to cluster with one another than the non-embryonated egg (Fig. 10c, d). Egg clumps may make microscopic evaluation difficult.

### Recovery of larvae from the lung

**Fig. 11:**
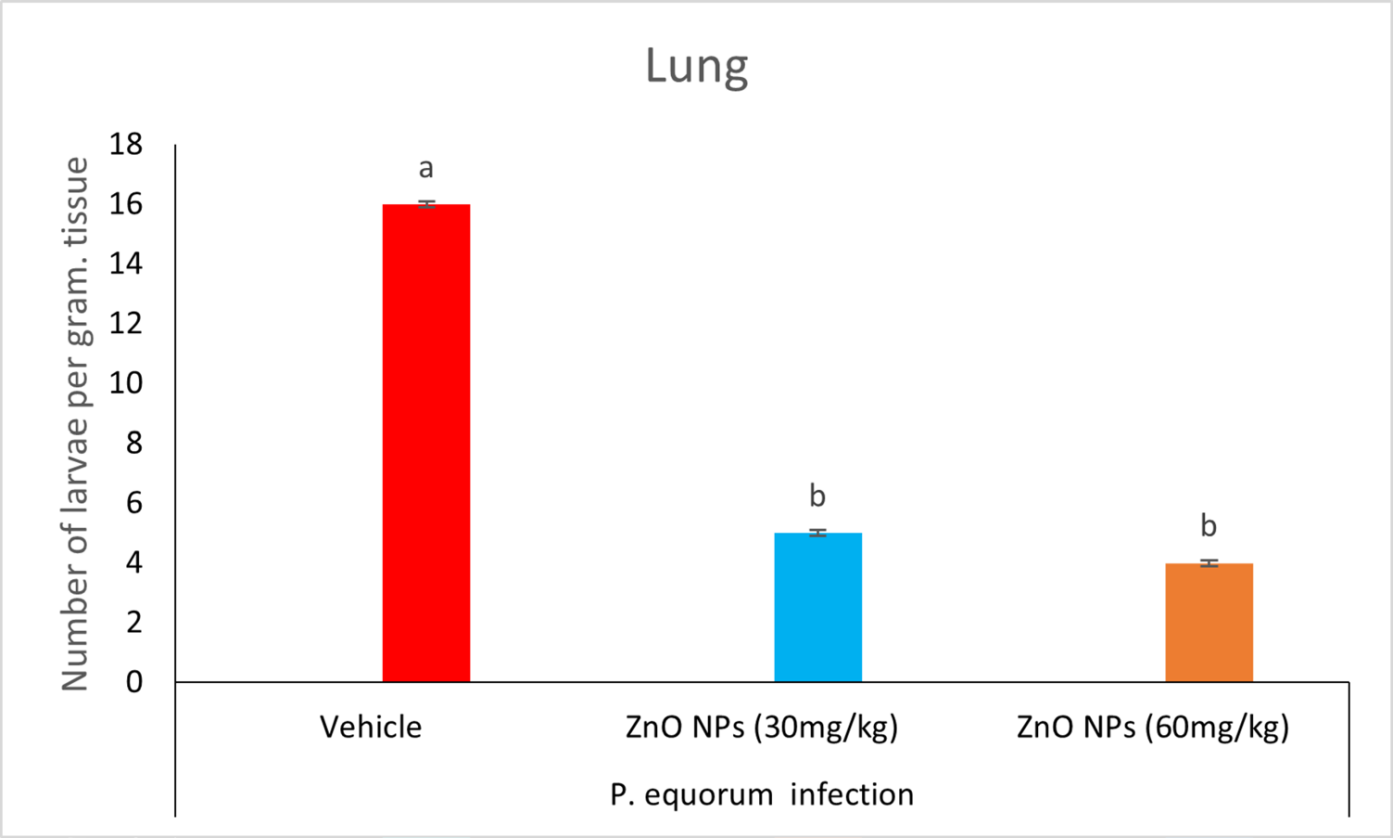
Counts of larvae in an infected lung.

**Fig. 12:**
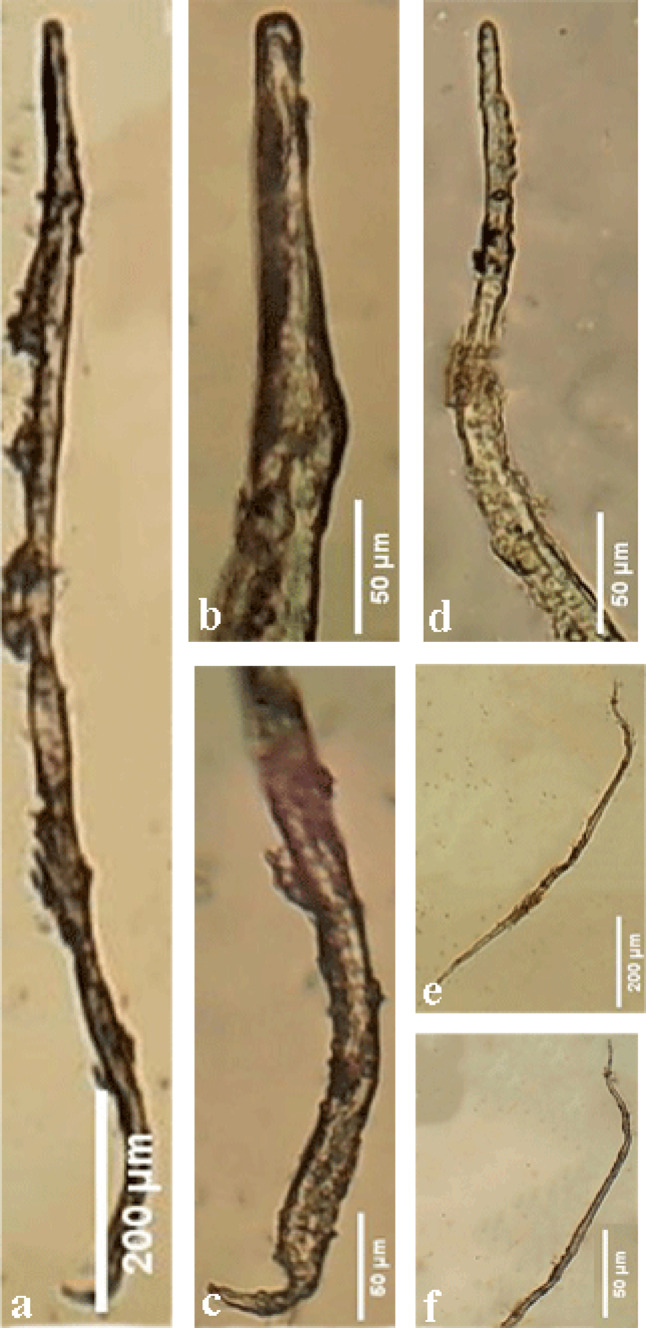
Photomicrographs of *P. equorum* larvae. **(a**,** b**,** c**) Recovered larvae from infected spleen (**d**,** e**,** f**). Recovered larvae from the infected lung.

There were sixteen larvae per gram of tissue in the vehicle group. Recovery of five larvae per gram of tissue was observed in the group treated with 30 mg/kg ZnO NPs. Additionally, the group treated with ZnO NPs (60 mg/kg) recovered four larvae per gram of tissue, in comparison to the untreated infected animals. The infected group that did not receive treatment with ZnO NPs (30, 60 mg/kg) exhibited a considerably reduced larval count (*P* < 0.05) in comparison to the treated group (Table 3) (Figs. 11 and 12).

### Recovery of larvae from the spleen

**Fig. 13:**
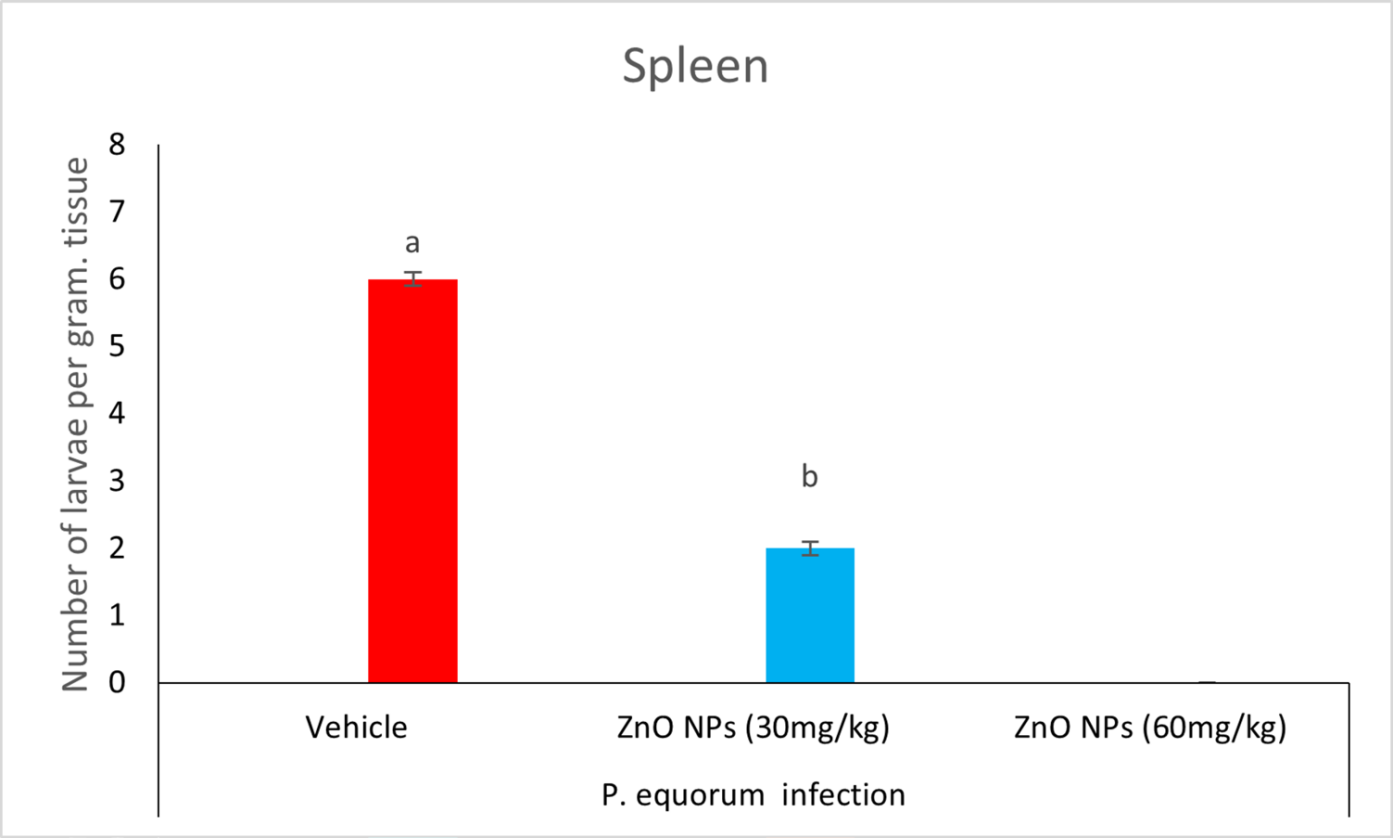
Counts of larvae in an infected spleen.

Recovery of six larvae per gram of tissue was observed in the vehicle group. The ZnO NPs-treated (30 mg/kg) group recovered two larvae per gram of tissue. In contrast, the 60 mg/kg group did not recover any larvae, in contrast to the untreated infected animals. The infected group that received treatment with ZnO NPs (30, 60 mg/kg) exhibited a significant reduction (*P* < 0.05) in larvae count when compared to the untreated group (Table 3) (Figs. 12 and 13).

### Scanning electron microscopy (SEM) examination of larvae from lung tissues

**Fig. 14:**
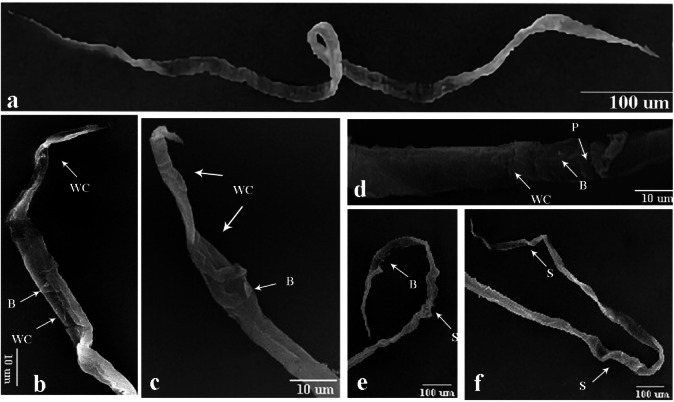
Scanning electron micrographs of *P. equorum* larvae (L3) from the infected lung. **(a)** Recovered larvae from infected untreated rats (**b**,** c**). Recovered larvae from infected rats treated with (30 mg/kg) ZnO NPs (**d**,** e**,** f**). Recovered larvae from infected rats treated with (60 mg/kg) ZnO NPs.

Figure 14a showed scanning electron micrographs of *P. equorum* larvae from untreated infected rats. Recovered larvae showed a normal appearance and a smooth body with no morphological or structural alterations. These larvae were poorly differentiated, with a tapered, rounded head. They displayed the typical coiling behavior of nematode larvae when removed from the host.

Scanning electron microscopy examination of the recovered larvae from rats treated with 30 mg/kg of ZnO NPs showed an altered morphological appearance with conspicuous structural changes than those from untreated infected rats. The classical shape of larvae was obscured as the anterior and posterior ends lost their typical rounded, tapered shape and became tortuous ends. Larvae exhibited many wrinkles cuticle (WC) along the anterior and posterior ends with some blebs (B) as compared to untreated larvae. In addition, the larvae recovered from infected treated rats were unable to exhibit the coiling activity characteristic of nematode larvae (Fig. 14b, c).

SEM examination of the recovered larvae from rats treated with ZnO NPs (60 mg/kg) showed profoundly injured regions. The anterior and posterior ends of the larvae became more flattened and thinner. They had wrinkled cuticle (WC) and shrunken (S) cuticle with small blebs (B) on the wall as compared to untreated larvae. Also, it had some pores (P) along the middle part of the larvae. Moreover, the larvae recovered from infected treated rats were unable to exhibit the coiling activity characteristic of nematode larvae (Fig. 14d, e, f).

### Oxidative stress characteristics of the lung and spleen

The MDA and NO concentrations of the vehicle group were significantly higher (*P* < 0.05) than those of the control group, according to data in Tables 1 and 2. On the other hand, when ZnO NPs were administered, MDA and NO concentrations were significantly (*P* < 0.05) lower than in the vehicle group.

**Table 1:** Therapeutic effect of ZnO NPs on lung oxidative stress markers in *P. equorum* infected rats.

Parameters Groups		MDA (nM/g.tissue)	GSH (mM/g.tissue)	CAT (U/min/g.tissue)	GST (µM/g.tissue/min)	NO (µM/g.tissue)
Noninfected groups	Control	1.89 ± 0.08	2.63 ± 0.23	231.08 ± 14.77	0.69 ± 0.08	538.12 ± 18.66
	ZnO NPs (30 mg/kg)	2.07 ± 0.30	2.39 ± 0.16	216.20 ± 4.67	0.59 ± 0.02	584.25 ± 17.29
	ZnO NPs (60 mg/kg)	1.95 ± 0.11	2.57 ± 0.04	229.57 ± 4.00	0.64 ± 0.03	544.43 ± 15.24
Infected groups	Vehicle	5.66 ± 0.12^a^	1.53 ± 0.12^a^	120.46 ± 6.42^a^	0.35 ± 0.01^a^	1,295.51 ± 11.66^a^
	ZnO NPs (30 mg/kg)	3.73 ± 0.15^b^	1.84 ± 0.15^b^	152.45 ± 16.29^b^	0.45 ± 0.03^b^	917.99 ± 17.66^b^
	ZnO NPs (60 mg/kg)	2.96 ± 0.30^b^	2.31 ± 0.12^b^	217.69 ± 18.22^b^	0.55 ± 0.03^b^	723.72 ± 14.14^b^

Results are expressed as mean ± SE in each group.

a: significant at (*P* < 0.05) compared to control group.

b: significant compared to vehicle group.

**Table 2:** Therapeutic effect of ZnO NPs on spleen oxidative stress markers in *P. equorum* infected rats.

Parameters Groups		MDA (nM/g.tissue)	GSH (mM/g.tissue)	CAT (U/min/g.tissue)	GST (µM/g.tissue/min)	NO (µM/g.tissue)
Noninfected groups	Control	3.85 ± 0.24	6.33 ± 0.08	179.30 ± 2.99	1.41 ± 0.02	3,337.94 ± 199.06
	ZnO NPs (30 mg/kg)	4.11 ± 0.15	5.75 ± 0.11	153.85 ± 7.77	1.33 ± 0.05	3,687.68 ± 64.19
	ZnO NPs (60 mg/kg)	3.99 ± 0.11	6.15 ± 0.10	167.92 ± 8.06	1.33 ± 0.10	3,414.70 ± 178.85
Infected groups	Vehicle	7.37 ± 0.12^a^	3.53 ± 0.14^a^	76.20 ± 1.82^a^	0.63 ± 0.03^a^	5,089.40 ± 203.11^a^
	ZnO NPs (30 mg/kg)	6.34 ± 0.04^b^	4.16 ± 0.08^b^	100.89 ± 3.24^b^	0.97 ± 0.04^b^	4,167.44 ± 129.65^b^
	ZnO NPs (60 mg/kg)	5.13 ± 0.05^b^	5.32 ± 0.20^b^	140.09 ± 4.53^b^	1.17 ± 0.02^b^	3,413.01 ± 107.59^b^

Results are expressed as mean ± SE in each group.

a: significant at (*P* < 0.05) compared to control group.

b: significant compared to vehicle group.

**Table 3:** Therapeutic effect of zinc oxide nanoparticles on larval count in the lungs and spleen of *P. equorum* infected rats.

Groups Organ	Vehicle	ZnO NPs (30 mg/kg)	ZnO NPs (60 mg/kg)
Lungs	16.2 ± 0.17^a^	4.8 ± 0.17^b^	5.2 ± 0.17^b^
Spleen	5.8 ± 0.17^a^	1.7 ± 0.05^b^	0 ± 0

Results are expressed as mean ± SE in each group.

a: significant at (*P* < 0.05) compared to control group.

b: significant compared to vehicle group.

Compared to the control group, the vehicle group’s GSH, CAT, and GST levels showed a substantial drop (*P* < 0.05). There was a noteworthy rise (*P* < 0.05) in the levels of GSH, CAT, and GST following the administration of ZnO NPs (30,60 mg/kg) in contrast to the vehicle group (Tables 1 and 2).

### Lung tissue histopathological analysis

**Fig. 15:**
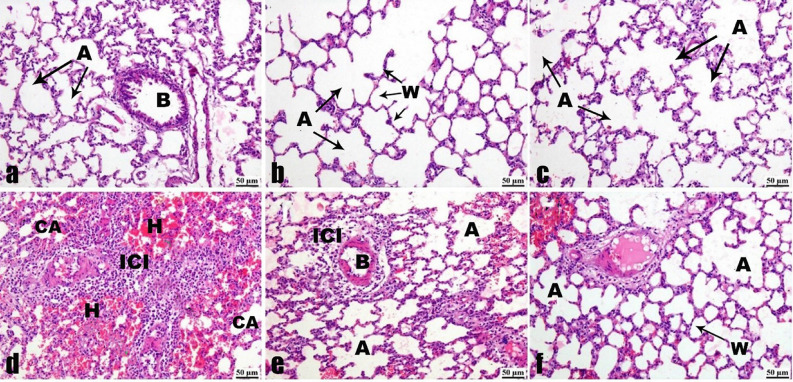
Photomicrographs of lung sections (H & E). (**a**) Control group, (**b**) 30 mg/Kg administered group, (**c**) 60 mg/Kg administered group. Standard histological structure of pulmonary alveoli (A), bronchioles (B), and thin interalveolar wall (W). (**d**) vehicle group exhibiting interstitial pneumonia characterized by inflammatory cell infiltrations (ICI), collapsed alveoli (CA), and interstitial hemorrhages (H). **(e)** The 30 mg/Kg treated group exhibited mild inflammatory cell infiltrations (ICI) and an improved alveolar space (A). (**f**) 60 mg/Kg treated group exhibiting pulmonary alveoli (A) relatively normal, and wall (W) thickness decreased.

Lung sections from the ZnO NPs-administered groups (at doses of 30 and 60 mg/Kg) and the control group showed a histologic structure of pulmonary alveoli and respiratory bronchioles that was within the normal range upon microscopic examination. The lung displayed a spongy structure with thin interalveolar walls and typical, clear alveoli. It was seen that the respiratory bronchioles were open and had a layer of single columnar epithelium covering them (Fig. 15a, b, c).

The lung sections obtained from untreated rats that were infected exhibited multifocal interstitial pneumonia characterized by inflammatory cell infiltration, increased aggregation of eosinophils, and interstitial hemorrhages. Furthermore, the walls of the alveoli were significantly thickened, and the alveoli collapsed as a result of excessive infiltration of white blood cells in the interalveolar septa (Fig. 15d).

The lung tissues of rats treated with a dosage of 30 mg/kg exhibited a decrease in the inflammatory reaction, characterized by slight perivascular edema and modest infiltration of inflammatory cells. The volume of the alveoli increased and the thickness of the walls reduced, approaching a normal state. (Fig. 15e). The lung tissues of rats treated with ZnO NPs at a dosage of 60 mg/kg exhibited the most significant decrease in the inflammatory response. The structure of lung tissue and pulmonary alveoli exhibited a generally normal appearance, with the alveolar space regaining its usual dimensions and shape, characterized by thin walls (Fig. 15f).

### Immune expression of NF-κβ in lung and spleen tissues

**Fig. 16:**
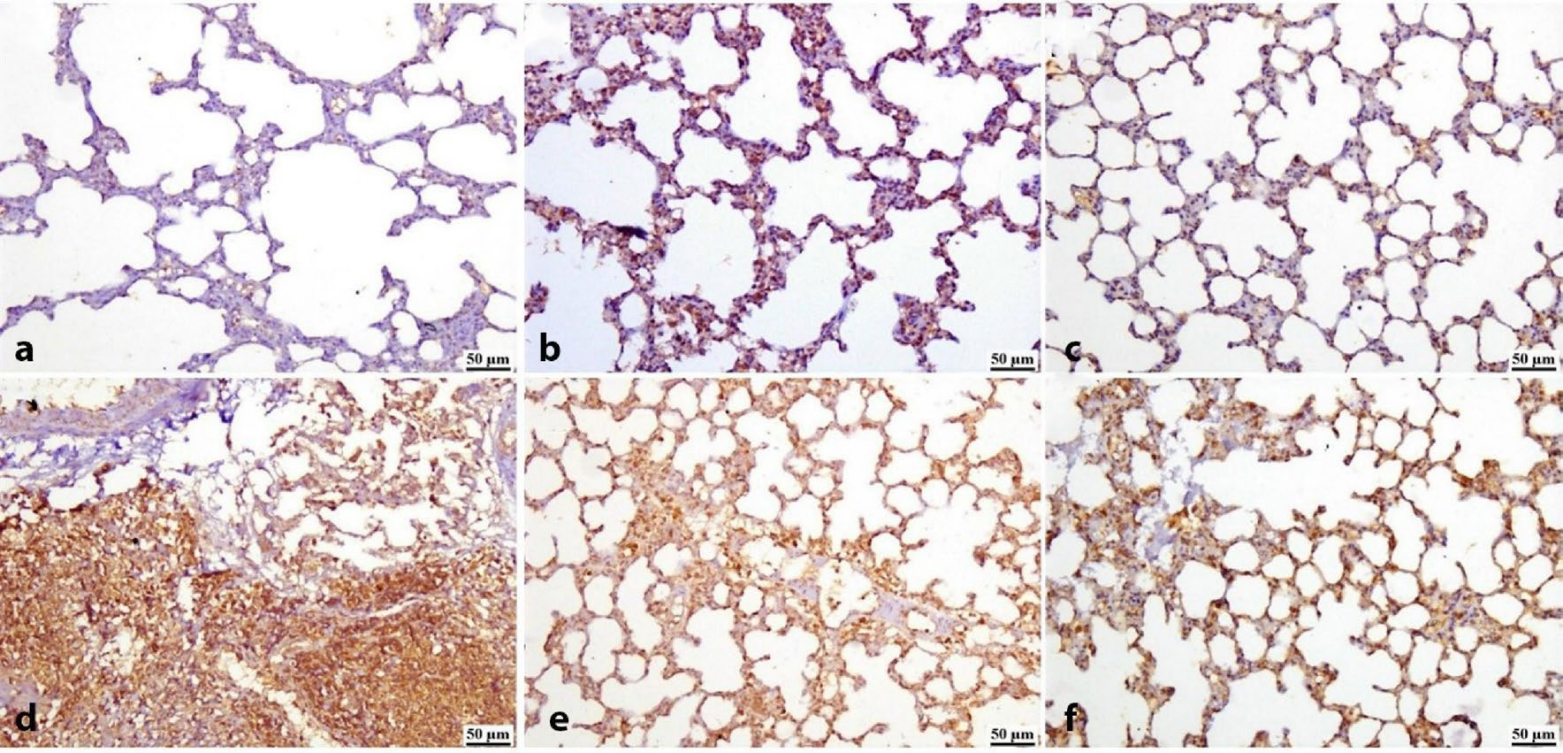
Photomicrographs of an immunostained lung. (**a**) control group, (**b**) 30 mg/Kg administered group, (**c**) 60 mg/Kg administered group exhibiting normal to limited NF-κβ expression, **(d)** vehicle group exhibiting a marked increase in NF-κβ expression. **(e)** 30 mg/Kg treated group and (**f**) 60 mg/Kg treated group showing a reduction in NF-κβ expression.

The data shown in Fig. 16 examined the expression of NF-κβ in lung tissues using immunohistochemistry. The results of the one-way ANOVA showed that the control group (Fig. 16a) had normal levels of NF-κβ expression, while the groups that received ZnO NPs (30, 60 mg/Kg) had normal levels of lung NF-κβ expression (Fig. 8b, c). In contrast to the control group, which showed regular expression of NF-κB in the respiratory bronchioles and pulmonary alveoli of the damaged lung tissues, the infected untreated group showed significant expression of the gene (Fig. 8d). Furthermore, following 10 days of ZnO NPs (30, 60 mg/kg body weight) treatment, a decrease in NF-κβ expression in the lung tissues of infected rats was seen in comparison to the infected non-treated rats (Fig. 16e, f).

**Fig. 17:**
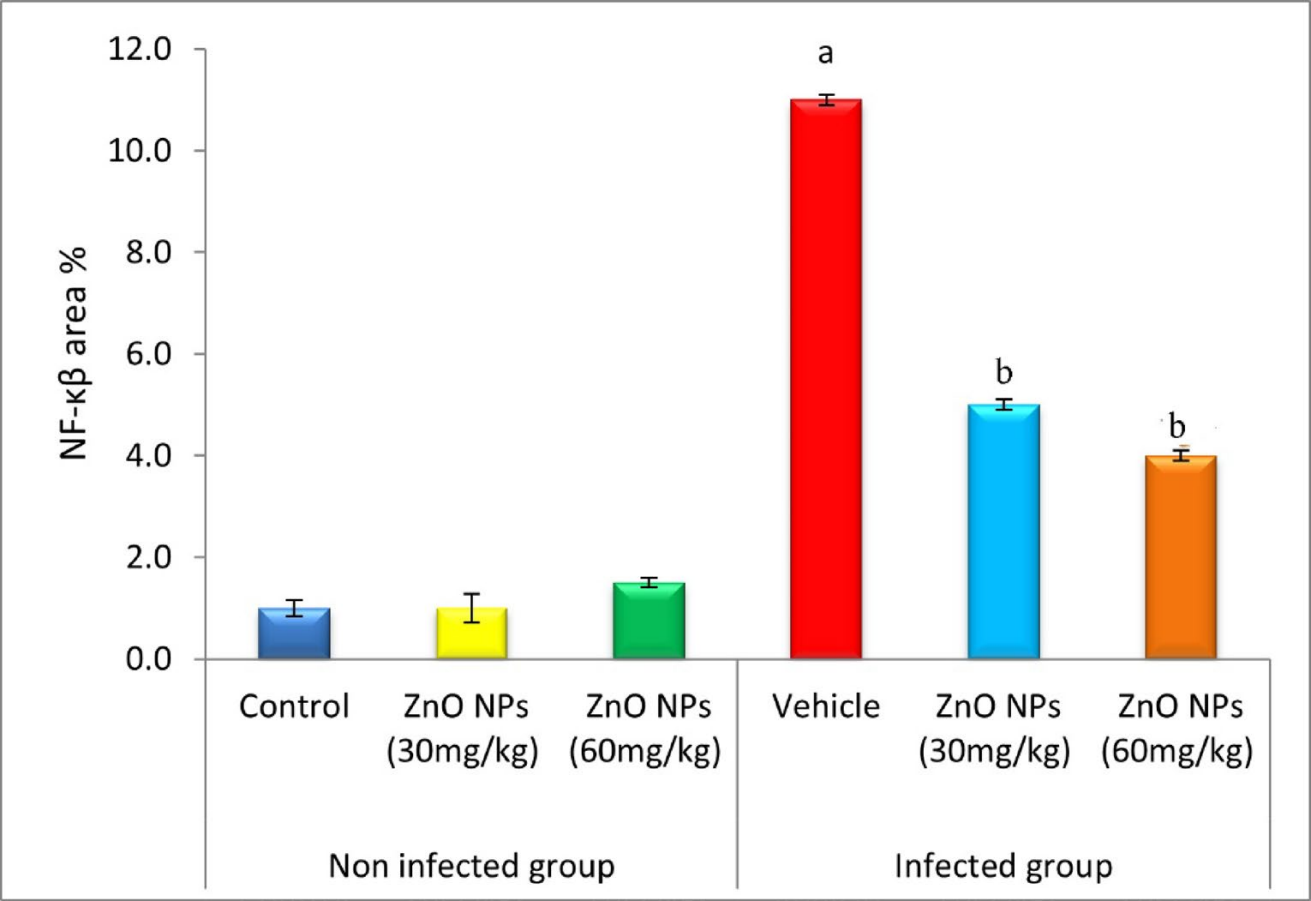
Therapeutic effect of ZnO NPs on NF-κβ expression in lung tissues of *P. equorum* infected rats. Results are expressed as mean ± SE in each group. (**a**): significant at (*P* < 0.05) compared to the control group. (**b**): significant compared to the vehicle group. (**c**): significant compared to ZnO NPs (30 mg/kg) treated group.

Figure 17 shows the quantification of NF-κβ in lung tissues. NF-κβ expression was significantly lower (*P* < 0.05) in the vehicle group and significantly greater (*P* < 0.05) in the ZnO NPs (30,60 mg/Kg) treated groups when compared to the control group. The ZnO NPs (30,60 mg/Kg) and the administered groups did not differ statistically significantly from the control group. However, following 10 days of ZnO NPs (30, 60 mg/kg) therapy, there was a substantial (*P* < 0.05) decrease in the expression of NF-κβ in infected rats compared to the infected rats that were not treated.

### Spleen histopathological examination

**Fig. 18:**
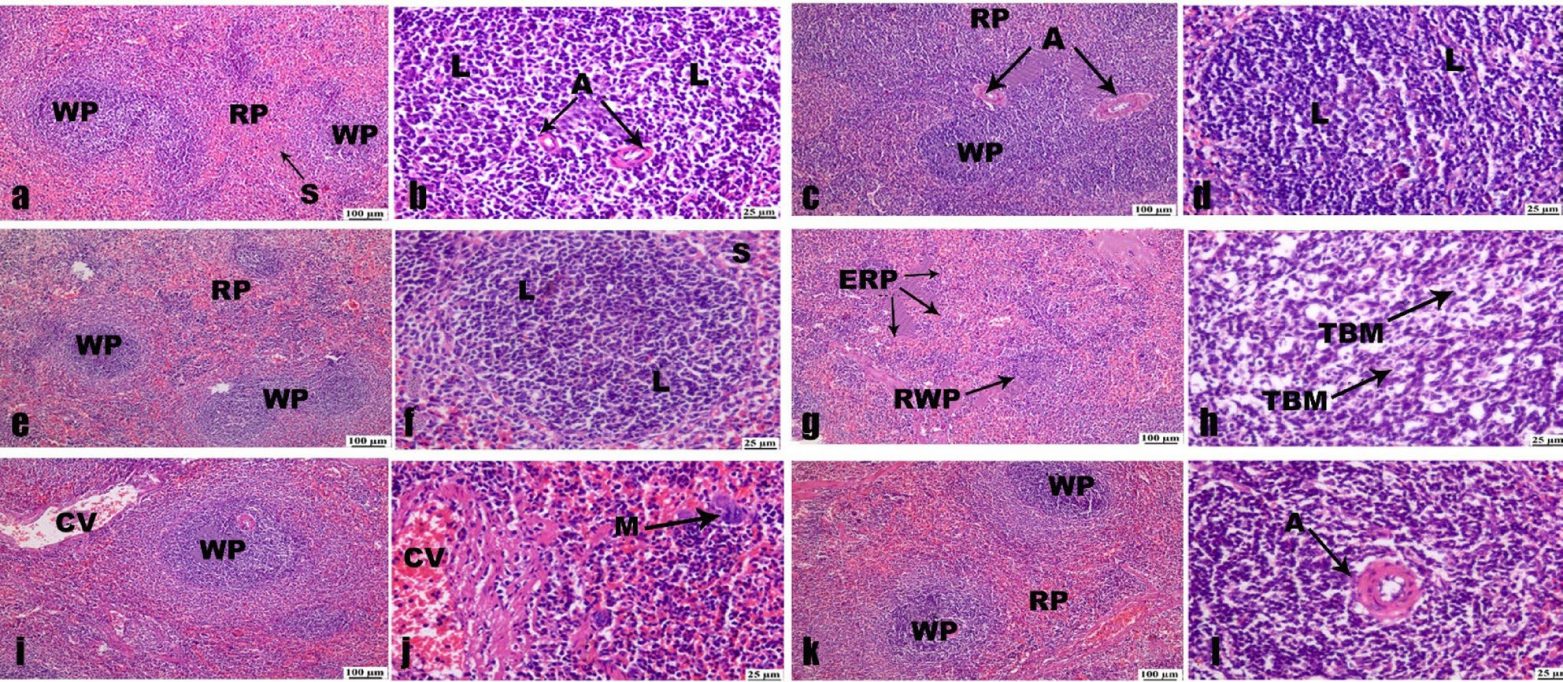
Photomicrographs of spleen sections (H&E). (**a**) and (**b**) control group, (**c**) and (**d**) 30 mg/Kg administrated group, (**e**) and (**f**) 60 mg/Kg administrated group, (**g**) and (**h**) vehicle group exhibiting expansion of splenic red pulps (ERP), reduction of splenic white pulp (RWP) and tingible body macrophages (TBM). (**i**) and (**j**) 30 mg/Kg treated group exhibiting normal and variable-sized white pulp (WP), congested blood vessels (CV), and a few megakaryocytes (M). (**k**) and (**l**) 60 mg/Kg treated group exhibiting normal splenic parenchyma in white and red pulps. Normal white pulp (WP) containing lymphocytes (L) and arteries (A), in addition to red pulp (RP) containing venous sinuses (S).

Microscopic analysis of splenic sections from the control (Fig. 18a, b) and ZnO NPs (30 and 60 mg/Kg) administered groups (Fig. 18c, d, e, f) showed that 80% of the splenic tissues are composed of normal parenchyma with white and red pulps. The splenic red pulp was made up of blood-filled venous sinuses and cords of lymphatic cells. In contrast, the splenic white pulp is lymphatic tissue primarily made up of lymphocytes surrounding the arteries.

The splenic architecture of infected, untreated rats’ splenic tissues showed significant histological anomalies. It was common to see certain splenic white pulps shrink and splenic red pulps expand. Apoptosis was observed in lymphoid follicles, and the tangible body macrophages (TBMs) that resulted from the engulfment of apoptotic bodies by macrophages. Primarily located in germinal centers, TBMs were a type of macrophage that included a large number of phagocytized apoptotic cells in varying degrees of disintegration (Fig. 18g, h).

On the splenic tissues of infected rats, the therapeutic impact of 30 mg/kg ZnO NPs demonstrated a modest level of protection. Several white pulps of varying sizes were present. The lymphoid follicles appeared to be normal (Fig. 18i). Additionally, in some regions with fewer megakaryocytes, mildly clogged blood arteries were seen (Fig. 18j). In contrast, the white and red pulps’ seemingly normal splenic parenchyma demonstrated the maximum degree of therapeutic protection when exposed to 60 mg/kg ZnO NPs (Fig. 18k, l).

## Discussion

Horses and donkeys are highly susceptible to the effects of the parasitic worm *Parascaris equorum* on their health, productivity, and overall well-being^17^. This drug can cause gastrointestinal harm, colic, pneumonia, and bronchial bleeding^18^. Anthelmintic medicines have excellent efficacy, but incidences of drug resistance have been well-documented^8^. Hence, nanomaterials offer a viable solution for treating many parasite illnesses that impact the well-being of both humans and animals, especially in combating drug resistance^19^. This study illuminates the healing properties of environmentally friendly zinc oxide nanoparticles on rats who are infected with *P. equorum*. In the current study, the non-embryonated eggs recovered from adult female worms were examined under the scanning electron microscope. In agreement with^20^, the non-embryonated eggshell had an appropriately smooth surface with ridges and plateaux. In addition, they had a strong tendency to cluster with one another and form egg clumps. Nematode eggs, particularly those isolated directly from the uteri of female worms, have a sticky proteinaceous coat, which increases the tendency of eggs to form clumps^21^.

After 20 days of in vitro egg embryonation, the eggs were examined by scanning electron microscope. In agreement with^22^, the surface of the embryonated eggshell appeared granular and devoid of plateaux and ridges, as shown in the non-embryonated eggs. In addition, embryonated eggs had a lower tendency to cluster with one another than non-embryonated ones. This may be because after egg embryonation, the larvae produce hatching enzymes such as proteases used in the degradation and thinning of the sticky proteinaceous coat, converting it to a fibrous protein coat^23^.

In the present investigation, ZnO NPs (30, 60 mg/kg) treatment resulted in a significant reduction in the number of larvae in the lung and spleen of *P. equorum*-infected rats, in a dose-dependent manner. Zinc oxide nanoparticles, or ZnO NPs, have a potent anthelmintic effect by harming worms’ cuticles, which are their outermost layer of defense^1^. The larval cuticle is harmed by metal nanoparticles when the positively charged metal ions that are released bond to the negatively charged cell membrane and disrupt its structural integrity. In addition, metal ions can also bind to the membrane wall, resulting in the formation of pores and openings through which they can enter the body^24^. Reactive oxygen species (ROS) are produced in excess by the body, and their neutralization by antioxidant defences is known as oxidative stress. Multiple severe disorders, including cancer, cardiovascular disease, neurological illness, diabetes, ischemia/reperfusion injuries, rheumatoid arthritis, and parasite infection, can result from oxidative stress damaging cells, tissues, and organs^25^.

An accepted mechanism of cellular damage, lipid peroxidation (LP), can be used to detect oxidative stress in tissues and cells. The complex process of lipid peroxidation (LP) is brought on by the interaction of reactive oxygen species with unsaturated fatty acids in cellular membranes, which damages the cells^26^. Since malondialdehyde (MDA) is the outcome of lipid peroxidation, estimating its level indirectly evaluates the degree of reactive oxygen species (ROS) and lipid peroxidation (LP)^27^. Furthermore, NO is a potent mediator that performs multiple roles in controlling cellular activities, lowering oxidative stress, and signaling both pathogenic and beneficial pathways^28^. Elevated concentrations of MDA (malondialdehyde) and NO (nitric oxide) have been linked to various chronic illnesses, such as parasite infections^29^.

The ongoing analysis revealed that the levels of MDA and NO in the lung and spleen tissues of rats infected with *P. equorum* had shown a rise. Intestinal parasites cause inflammation, reactive oxygen species (ROS) to be produced, and naturally existing antioxidants to decline^30^. Reactive oxygen species (ROS) are made too much in the metabolic pathway, which raises lipid peroxides, especially malondialdehyde (MDA), the main product of lipid peroxidation^31^. Recent research suggests that helminth infection induces macrophages to generate nitric oxide (NO), which then promotes the progression of the disease. Furthermore, parasites can generate nitric oxide (NO) as a means of defending themselves against the immunological response of the host^32^. Rats infected with *P. equorum* had considerably lower levels of nitric oxide (NO) and malondialdehyde (MDA) in their spleen and lung tissues when zinc oxide nanoparticles were applied. Zinc oxide nanoparticles (ZnO NPs) can operate via a variety of antioxidant pathways, such as lipid peroxidation inhibition and the scavenging of active oxygen radicals^33^. Moreover, nitric oxide (NO) and its oxidized counterpart, peroxynitrite, are produced, and both aberrant states can be managed by nanoparticles^8^. By averting oxidative stress and cellular damage, antioxidant substances mitigate the deleterious consequences of free radicals^34^. The body naturally produces glutathione (GSH), glutathione peroxidase, catalase (CAT), alpha-lipoic acid, and superoxide dismutase (SOD). Exogenous antioxidants include vitamins C, E, beta-carotene, selenium, bioflavonoids, and N-acetyl cysteine (NAC)^35^. In the lung and spleen tissues of rats infected with *P. equorum*, the present study showed a decrease in glutathione (GSH) levels, catalase (CAT), and glutathione S-transferase (GST) activity. Intestinal parasites impair the antioxidant system’s ability to operate by lowering GSH levels, CAT and GST enzyme activity, and other parameters^36^. The decrease was attributed to increased cytotoxicity brought on by H2O2 because glutathione reductase, which keeps glutathione in its reduced state, is suppressed by it.

Furthermore, in the process of being neutralized, the host’s stores of glutathione (GSH), catalase (CAT), and superoxide dismutase (SOD) are depleted by the reactive oxygen species (ROS) that the parasites produce^32^. Reduced GSH inhibits glutathione peroxidase activity and can cause dangerous quantities of lipid peroxidation products to accumulate^37^. GSH is a co-substrate for glutathione peroxidase. On the other hand, rats treated with ZnO NPs after contracting *P. equorum* infection showed a significant increase in CAT, GST, and GSH levels in their lung and spleen tissues. ZnO nanoparticles’ anti-inflammatory and antioxidant properties protect the cell membrane from reactive oxygen species (ROS)-induced damage and promote the synthesis of detoxifying and antioxidant enzymes^38^.

Based on lung and spleen histology, the current investigation revealed that rats infected with *P. equorum* had considerable pulmonary hemorrhages and inflammation. As the larvae pass through the lung tissues, mononuclear cells infiltrate the area, causing pulmonary lesions, bronchial epithelial deterioration, and airway cell shedding^39^. Moreover, the current study revealed that *P. equorum* infection in rats resulted in significant histological alterations to the splenic architecture. At the location of infection, the migration of nematode larvae causes an inflammatory response characterized by neutrophil infiltration, splenic red pulp expansion, and shrinkage of certain white pulps^40^. On the other hand, ZnO NPs reduced the amount of inflammation and bleeding in the lungs and spleen of *P. equorum*-infected rats. According to^6^, zinc oxide nanoparticles (ZnO NPs) have anthelmintic activity against larvae as well as antioxidant and anti-inflammatory qualities that strengthen the tissues of the spleen and lungs’ resistance to larval migration. According to immunohistochemistry, a transcription factor (TF) is a protein that regulates the rate at which genetic information is converted from DNA to messenger RNA. By attaching to a specific DNA sequence, it accomplishes this^41^. Nuclear factor-kappa B (NF-κB) is a transcription factor that has a crucial function in innate immunity and the ability to fight off infections. It stimulates the activation of genes that produce pro-inflammatory cytokines and controls the development and viability of both innate immune cells and lymphocytes^42^. The NF-κB pathway significantly influences the initiation of an immune response. But several pathogens—including bacteria, viruses, and parasites—have evolved a number of tactics to obstruct this process in order to further their own interests^43^. Pathogens or their constituents exhibit a notable ability to interfere with the NF-κB pathway at multiple levels, encompassing the receptors that are membrane-bound and the signaling molecules that are downstream of the process^42^. The present study showed that NF-κβ was strongly expressed in the lung tissue of rats infected with *P. equorum*. When a pathogen infects a host cell, the host’s NF-κB signaling pathways are usually activated, which causes NF-κB production to be upregulated. A protective regulatory response is induced by NF-κB^42^. However, ZnO NP administration reduced the amount of NF-κβ expressed strongly in lung tissue, indicating that ZnO NPs have anthelmintic effects on *P. equorum* larvae in rats. A histological study showed that the spleens of rats infected with parasites had only minor tissue damage. As a result, NF-κB expression was low and hard to find in the spleen tissues.

## Conclusion

In the current study, administration of green-synthesized ZnO NPs decreased the number of *P. equorum* larvae in treated infected rats. In addition, it improved oxidative stress status by decreasing MDA and NO levels and increasing antioxidant markers such as GSH content and GST and CAT activities in *P. equorum*-infected rats, indicating that ZnO NPs can act through many antioxidant mechanisms, including the scavenging of active oxygen radicals and inhibition of lipid peroxidation. Moreover, immunohistochemical analysis of nuclear factor-kappa B showed a significant decrease during the treatment with ZnO NPs (30, 60 mg/kg) compared to the infected untreated animals, indicating the anthelmintic action of ZnO NPs against *P. equorum* infection in rats. Consequently, we suggested green-synthesized ZnO NPs as a potential alternative anthelmintic agent in parasitic diseases.

## Data Availability

The datasets of the current study are available from the corresponding author on a reasonable request.
